# Sensory Information Modulates Voluntary Movement in an Individual with a Clinically Motor- and Sensory-Complete Spinal Cord Injury: A Case Report

**DOI:** 10.3390/jcm12216875

**Published:** 2023-10-31

**Authors:** Claudia Angeli, Sarah Wagers, Susan Harkema, Enrico Rejc

**Affiliations:** 1 Tim and Caroline Reynolds Center for Spinal Stimulation, Kessler Foundation, West Orange, NJ 07052, USA; enrico.rejc@louisville.edu; 2 Department of Bioengineering, University of Louisville, Louisville, KY 40292, USA; 3 Kentucky Spinal Cord Injury Research Center, University of Louisville, Louisville, KY 40202, USA; sarah.wagers@louisville.edu (S.W.); susan.harkema@louisville.edu (S.H.); 4 Division of Physical Medicine and Rehabilitation, University of Louisville, Louisville, KY 40292, USA; 5 Department of Medicine, University of Udine, 33100 Udine, Italy

**Keywords:** spinal cord injury, clinically motor and sensory complete, neurorehabilitation, voluntary movement, sensory information

## Abstract

Motor recovery following a complete spinal cord injury is not likely. This is partially due to insurance limitations. Rehabilitation strategies for individuals with this type of severe injury focus on the compensation for the activities of daily living in the home and community and not on the restoration of function. With limited time in therapies, the initial goals must focus on getting the patient home safely without the expectation of recovery of voluntary movement below the level of injury. In this study, we report a case of an individual with a chronic, cervical (C3)-level clinically motor- and sensory-complete injury who was able to perform voluntary movements with both upper and lower extremities when positioned in a sensory-rich environment conducive to the specific motor task. We show how he is able to intentionally perform push-ups, trunk extensions and leg presses only when appropriate sensory information is available to the spinal circuitry. These data show that the human spinal circuitry, even in the absence of clinically detectable supraspinal input, can generate motor patterns effective for the execution of various upper and lower extremity tasks, only when appropriate sensory information is present. Neurorehabilitation in the right sensory–motor environment that can promote partial recovery of voluntary movements below the level of injury, even in individuals diagnosed with a clinically motor-complete spinal cord injury.

## 1. Introduction

The International Standards for Neurological Classification of Spinal Cord Injury (ISNCSCI) has been used as the gold standard to assess and document the level and severity of an injury in spinal cord injuries (SCIs). Those classified with a grade A (AIS-A) have no motor or sensory function below the level of injury, while a classification of AIS-B implies impaired sensory function with no motor function below the level of injury. Motor- and sensory-incomplete injuries are classified as AIS-C and AIS-D. The AIS-D classification implies the greatest level of function with more than half the muscles below the level of injury and as having the ability to move against gravity, while an AIS-E is considered normal motor and sensory function. Following the classification of a motor-complete SCI, all volitional control of movement below the level of injury is theorized to be impossible. This clinical classification brings a substantial negative connotation to the capacity for rehabilitation and recovery following injury. Even with the increased evidence of plasticity of the spinal cord circuitry below the level of injury [1,2,3,4,5,6,7,8,9,10], a motor-complete classification leads all clinical interventions to be focused on compensatory strategies developed with the goal of integrating the individuals back into the home and community, disregarding the restoration of function. Only 19.3% of individuals classified with an AIS- A motor-complete injury restore their function after one year post-injury [11]. Similarly, 10% of AIS-A individuals convert to AIS B and an additional 10% regaining some motor function when converting to AIS C within the first year of injury [12]. Normal pinprick sensation at S1 over lateral heels within a month of the injury appears to be a good indicator of walking recovery at one year since a SCI [13]. Cervical injuries have a greater likelihood of AIS grade conversion when compared to thoracic injuries. Up to 77% of those that recover at least two grades in the AIS scale regain function within the first 3 months of injury [12]. In a prospective study to determine the motor recovery in patients with AIS-A, Fisher et al. (2005) [14] concluded that motor recovery distal to the injury does not occur in individuals with a motor-complete SCI. In a five-year study, Kirshblum et al. only found a 1% conversion from a motor-complete (AIS A) to motor-incomplete (AIS C) SCI [15]. They found no evidence of motor recovery in the lower extremities in individuals with tetraplegia.

Studies have shown that, even in individuals without volitional control of movement below the level of injury, reinforcement maneuvers can uncover supraspinal influence and promote non-specific activation of muscles below the level of injury [16,17,18]. Regardless of this observation, the integration into activity-based rehabilitation programs for individuals with a motor-complete SCI remains limited. In this report, we will show the capacity to generate and modulate motor output in an individual with a chronic motor-complete SCI and the role of sensory information in the successful execution of voluntary movement.

## 2. Detailed Case Description

A 31-year-old male with a C3 AIS-A SCI was screened for potential participation in a research study. The individual provided written informed consent for the electrophysiological assessments performed under the screening protocol (07-0224) approved by the University of Louisville’s Institutional Review Board. The individual sustained a traumatic SCI playing basketball 2.4 years prior to the assessments. At the time of injury, the emergency room examination revealed a sensory- and motor-level SCI of C4 without sacral sparing and no bulcocavernosus reflex. He underwent emergency surgery for decompression and stabilization of the C3-4 level. He was transferred to a medical intensive care unit for 6 weeks, followed by 2 months of inpatient rehabilitation in a dedicated spinal cord injury rehabilitation unit. He received a standard of care physical and occupational therapy focused on activities of daily living and home reintegration. An indwelling suprapubic catheter was selected for bladder management. As part of his outpatient rehabilitation program, the individual had undergone one year of intense activity-based recovery training at a community and fitness center specialized in SCIs near his hometown [19]. Activity-based recovery training is focused on tasks that activate the neuromuscular system below the level of injury with the goal of functional recovery of certain tasks. Activity-based recovery training exercises promote neuroplasticity through high-intensity, task-specific training with sensory stimulation [20]. Two key characteristics of activity-based recovery interventions are task-specific motor activation and sensory stimulation (appropriate sensory motor therapy) [21]. Task-specific sensory input provided to the spinal networks is critical in the activity-dependent plasticity and functional recovery [22].

Clinical evaluation revealed no ability to use upper extremities and no voluntary movement of the lower extremities when placed in a supine, sensory-deprived position. The ISNCSCI revealed an upper extremity score of 0 for both right and left sides and a lower extremity score of 0 for both right and left sides. Sensory light touch scores were 6 and 8 for the right and left sides, respectively, and pin prick scores were 4 and 5 for right and left sides, respectively. In addition, the ISNCSCI exam revealed an absence of motor and sensory function in the most caudal sacral segment (Appendix S1). The physical examination revealed hypertonicity, 3 or higher in the Ashworth scale, of hip extensors, knee flexors, hip adductors and ankle plantar flexors. Reflexes were hyper-responsive bilaterally for the patellar tendon and normal bilaterally for the Achilles tendon. The individual did not take anti-spasticity medications at the time of the assessments. The individual reports taking anti-spasticity medications for 5 months following injury, and, due to not liking the feeling of tiredness, he weaned off the medication. Some reinforcement maneuvers performed during a functional neurophysiological assessment elicited a long-lasting activation of the muscles below the level of injury [23].

In this case report, the individual performed a series of exercises on a mat at various positions and with different components of sensory information. Appropriate sensory input to the spinal networks was maximized in some tasks by promoting optimal load and kinematics through a full range of motion. These activities were routine practice through the activity-based recovery training program at the community and fitness center in his hometown. We collected electromyography (EMG) from bilateral lower extremity, trunk and upper extremity muscles using surface and fine-wire electrodes. We recorded EMG and kinematic data at 2000 Hz using a custom-written acquisition software (National Instruments, Austin, TX, USA). Joint kinematics of trunk, upper and lower extremities were recorded using a high-speed passive marker motion capture system (Motion Analysis, Santa Rosa, CA, USA). A mini-shuttle leg press (Shuttle Systems, Bellingham, WA, USA) was used during some knee extension attempts to offer resistance against the movement. For some assessments, joint torque was collected using a Biodex dynamometer (Biodex Medical Systems, Shirley, NY, USA). Table 1 describes the movements attempted and any variations performed. Most movements were attempted with appropriate sensory information, maximizing load and kinematics, and with suboptimal sensory information, in which load and/or kinematics were not optimal (Supplemental Video). Assessments include movements of the upper extremities, trunk and lower extremities.

**Figure 1:**
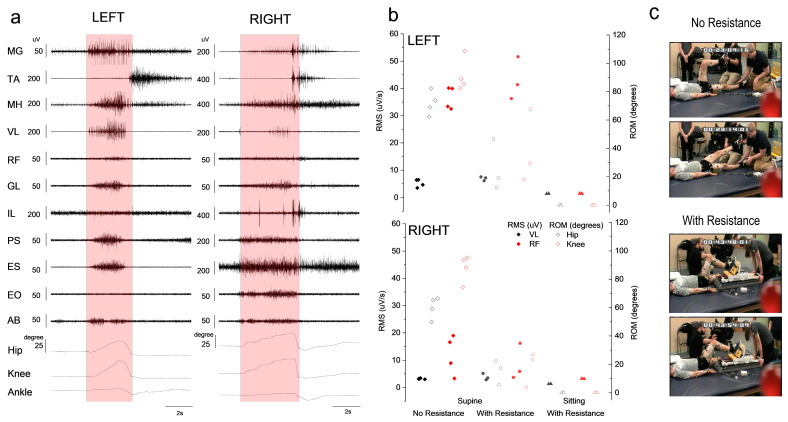
Quantification of motor output during knee extension exercise. (**a**) EMG activity of lower extremity and trunk muscles during single-leg knee extension using a mini-shuttle leg press from a supine position. Left panel: left side muscles and kinematics of the hip, knee and ankle during single-left-leg press. Right panel: right side muscles and kinematics during single-right-leg press. Mini-shuttle had 3 black and 1 red resistance bands connected. Red box represents the active portion of the exercise. Muscles: medial gastrocnemius (MG); tibialis anterior (TA); medial hamstrings (MH); vastus lateralis (VL); rectus femoris (RF); gluteus maximus (GL); iliopsoas (IL); paraspinal at T12 (PS); erector spinae (ES); external oblique (EO); rectus abdominus (AB). (**b**) RMS of vastus lateralis (black) and rectus femoris (red) muscles during multiple knee extension attempts. Diamonds (◊) represent attempts from a supine position without resistance; circles (○) are attempts from supine position using a mini-shuttle leg press (with resistance); triangles (Δ) are attempts from sitting position using a mini-shuttle leg press (with resistance). All open symbols represent the range of motion (ROM) of the knee (black) and hip (red) joints for the matching muscle data. Left (top) and right (bottom) data are shown in separate panels. (**c**) Photographs of the participant conducting single-leg extension without resistance (top) and with resistance (bottom).

We performed a comparison of hip and knee extension attempts from a supine position and from a sitting position. In both positions, the mini-shuttle was used to increase the resistance against the movement. A comparison of the mini-shuttle with and without resistance was also performed. Extensors of the ankle, knee, hip and low back were all activated during the active phase of the movement in both supine conditions (without and with resistance). Figure 1a shows a representative attempt (one on each side) for single-leg hip and knee extension against resistance from a supine position. In both cases the individual was able to move the mini-shuttle leg press against a load of 20lb and 24.5lb for the left and right sides, respectively. The root mean square (RMS) result for the hip extensor (GL) and knee extensor (VL) shows similar values for the movement with no resistance and against resistance (Figure 1b), although the range of motion about the hip and knee are greater without resistance. Load and kinematics promote appropriate sensory information throughout the task. RMS values during knee extension from the sitting position are negligible as the movement was unsuccessful. Knee extension was also repeated while sitting on the Biodex chair, which resulted in no muscle activation and no generation of torque during these attempts.

**Figure 2:**
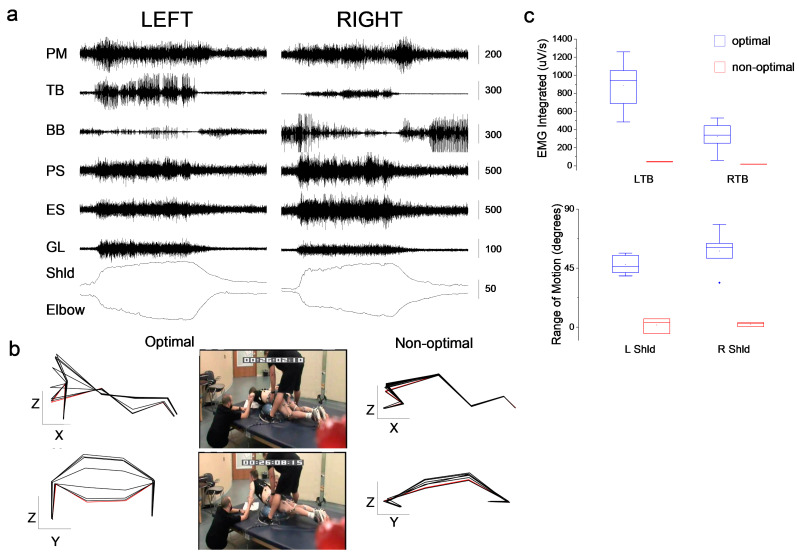
Quantification of motor output during a push-up exercise. (**a**) Bilateral EMG activity of upper extremity and trunk muscles during a push-up. Left panel: left side muscles and kinematics of the shoulder (Shld) and elbow; right panel shows right side muscles and kinematics. Muscles: pectoralis major (PM); triceps brachii (TB); biceps brachii (BB); paraspinal at T12 (PS); erector spinae (ES); gluteus maximus (GL). (**b**) Two-dimensional positional kinematic representation of an optimal (elbows supported wide) and non-optimal (elbows pointing posteriorly) push-up positions. A sagittal (X-Z) and frontal (Y-Z) views are shown for both variations. The non-optimal position resulted in an unsuccessful push-up. Photographs represent an optimal attempt. (**c**) Box plot of integrated EMG for the left triceps brachii (LTB) and right triceps brachii (RTB) during optimal push-up trials (blue box) and non-optimal push-up trials (red-box). Data are from 5 optimal attempts and 3 non-optimal attempts. The right panel is the range of motion of the shoulder for the same data set.

Figure 2 shows EMG modulation during a push-up performed by the research participant. When positioned to maximize appropriate sensory information, with palms of the hands on the mat, elbows supported in the frontal plane and hips on a Bosu ball, the individual was able to perform a full push-up, activating bilateral pectoralis major, triceps brachii and back and hip extensor muscles during the up phase (Figure 2a). The biceps brachii were activated following the down phase and during the return to the initial position. A comparison of integrated EMG of the triceps brachii during multiple repetitions in optimal (appropriate sensory information) and non-optimal (missed aligned upper extremities) positions show the inability to generate a sufficient burst in the elbow extensors to produce the desired movement (Figure 2b). The left and right mean integrated EMG differs, although the range of motion about the shoulder is similar. Attempts to perform shoulder flexion from a supine position deprived of all sensory information generated no EMG modulation in any of the muscles that were tested, resulting in an unsuccessful attempt of movement.

**Figure 3:**
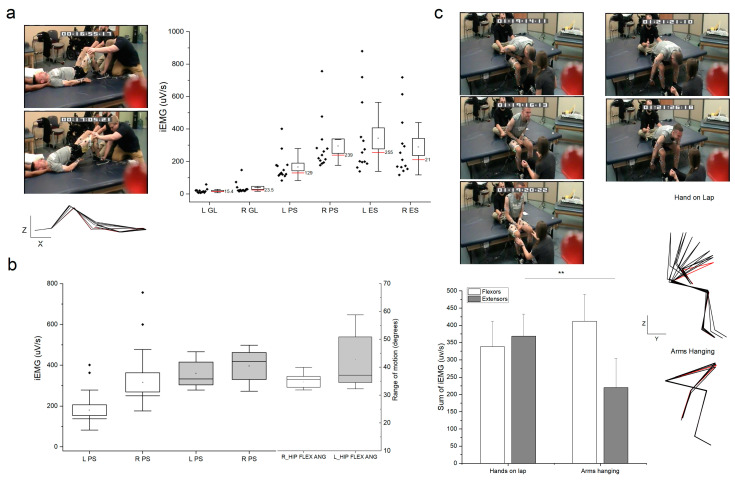
Quantification of motor output during trunk exercises. (**a**) Box plots of integrated EMG (iEMG) for left (L) and right (R) hip extensors and trunk extensors during a hip bridge exercise. Individual data points are shown to the left of box plot. Stick figure is the representative two-dimension kinematic data in the sagittal plane (X-Z) for one attempt. First frame is shown in red. Muscles: paraspinal at T12 (PS); erector spinae (ES); gluteus maximus (GL). (**b**) Box plots of integrated EMG (iEMG) for left (L) and right (R) paraspinal muscles during hip bridge exercise performed on a mat (white) and on a Biodex (Gray), data also show range of motion about the hip (flexion/extension) for both positions. (**c**) Bar graph representing the average and SE of the sum of integrated EMG for upper, lower extremity and trunk flexor muscles (white) and upper, lower extremity and trunk extensor muscles (gray) during two different trunk extension exercises. Each exercise was performed three times. Symbol ** denotes significant difference *p* < 0.005. The successful attempts had an initial position with upper extremities resting on thighs with the trunk fully flexed forward (photographs to the left and top stick figure). Initial position is shown in red. The unsuccessful attempts had an initial position with upper extremities hanging to the side of the legs with the trunk fully flexed forward (photograph to the right and bottom stick figure). Initial position shown in red.

Hip bridges were successful both on the mat and on the Biodex with the chair positioned fully reclined. Integrated EMG for hip and back extensors is shown in Figure 3a. Bilateral paraspinals and erector spinae are both active through the active phase of the movement, and, to a lesser extent, the gluteus maximus is also active. Box plots for the data of 13 repetitions during supine mat attempts show the range of activation across the muscles. When comparing the paraspinal activation on the mat to the activation during the Biodex assessment performed on a separate day, integrated EMG values are comparable between both, showing a slightly higher symmetry between the left and right sides during the Biodex attempts (n = 3). The range of motion for the hip joint was slightly greater, and more variable when hip bridges were performed on the Biodex (Figure 3b). A single event was successful to measure torque during the hip bridge attempt. The individual was able to generate a peak torque of 51.5 Nm about the hip joint during the successful attempt. Integrated EMG for the paraspinals in the torque assessment was comparable with the values seen during the range of motion assessment attempts.

Trunk extensions performed, starting with hands on lap, provided appropriate sensory information for the individual to successfully move from a fully bent forward position (full fold) to a tall sitting position. Figure 3c compares the sum of integrated EMG of the flexor muscles with the sum of integrated EMG of the extensor muscles (upper, lower extremity and trunk). The comparison also shows the difference between successful and unsuccessful attempts. All attempts performed with arms hanging to the side of the legs and without the additional sensory information from the upper extremities placed on the individual’s lap were unsuccessful for trunk extension. As shown, there was a significant difference between the total activation of extensor muscles during successful (greater activity) and unsuccessful (lower activity) events (*p* < 0.005).

## 3. Discussion

This individual, although classified with a C3 AIS-A SCI, was able to voluntarily control certain movements when placed in a sensory-rich environment. The individual was receiving intense activity-based training at a community and fitness center specialized in SCIs near his home town [24]. His daily training strategy was focused on the activation of the neuromuscular system below the level of injury, resulting in the restoration of motor control of upper and lower extremity muscles. Based on his reported abilities, we proceeded to quantify the modulation of his motor outputs during voluntary tasks. Evidence of neuroplasticity promoting the recovery of function following a spinal cord injury has been the focus of research for decades [22,25,26,27,28,29,30,31,32,33,34,35]. In the reported example, the individual was able to recover the ability to perform push-ups, trunk extensions and knee extensions by modulating motor activity below the level of injury when positioned in a sensory-rich environment with an appropriate mechanical advantage.

The role of sensory information has long been reported, more specifically, relative to locomotion [36,37,38]. However, the same principles can be applied to any movement. For this individual, an appropriate alignment of joints, providing a more mechanical advantageous position, were necessary for him to perform a push-up. A proper alignment and a wider base of support allowed for loading through the hands, enabling force transfer through the kinetic chain and the activation of the appropriate extensor muscles through an anti-gravity range of motion. During the push-up phase, the individual was able to activate not only his elbow extensors and shoulder adductors but also his trunk and hip extensors, showing appropriate motor control for the task. When elbows were not supported in a wide position and aligned with the hands, the individual was unsuccessful at generating an appropriate activation of extensor muscles. Hand position affects the forces about the elbow joint and muscle recruitment about the shoulder joint [39]. This loss of a mechanical advantage could partly explain the inability to successfully perform a push-up with unsupported elbows. However, the muscle activation was not sufficient to initiate movement in the non-optimal position. When shoulder flexion and elbow flexion were attempted from a supine position, the individual was unable to activate any upper extremity muscles, resulting in no range of motion about the joints. In the ISNCSCI scale, the individual was graded 0 for elbow extension, which correlated with the ineffective attempts to move the upper extremities when in a supine position.

Similarly to the push-up, the activation of extensor muscle groups generating a range of motion in an anti-gravity position could be demonstrated for the trunk. The individual was able to perform hip bridges when positioned supine with knees flexed, as well as trunk extensions when his upper extremities were resting on his lap. In both of these exercises, the individual properly activated trunk and hip extensors through a full range of motion. When performing trunk extensions, this task was only successful when his hands where resting on his lap, generating sensory information in the form of loading through the arms which was used to aid in the extension movement. When comparing the activation of the flexors and extensors between the trunk extensions with sensory feedback and without sensory feedback (arms hanging), the flexors were predominantly active in the unsuccessful attempts, when compared to the extensors in the successful attempts.

Although the focus of this case report is to bring attention to the neuroplasticity that is available even in SCIs classified as an AIS-A, the implications of the reported observations are widespread. With the advancement of neuromodulation and positive outcomes obtained with epidural stimulation in individuals with motor complete injuries [40,41,42,43,44,45,46], greater attention and opportunities should be provided to individuals with AIS-A by rehabilitation services and insurance providers. The ability to pay for rehabilitation remains a large limitation for equal access to activity-based services across all injury severities, provided that those classified with a motor-complete SPI typically only have access to these services through self-pay or scholarship opportunities [47].

The role of spasticity in motor recovery remains unclear [45,48]. However, a recent study suggests that spasticity is a physiological biomarker for the prediction of motor recovery in the subacute phase of SCI recovery [49]. In this case report, hypertonicity was reported clinically through the Ashworth scale. However, the ability to effectively modulate muscle tone to generate movement in certain positions reflects a positive outcome. In spite of that, a gap remains between the outcomes recorded and the ability to translate those to an independent life-style. However, the opportunity to remain engaged in a successful exercise program can have health and mental benefits [50]. These reports are analogous to individuals that might only receive exoskeleton therapy in clinics, and still report physiological and mental benefits from participating in this form of therapy, although this is not directly translated to improvements in the home and community [51]. It has been reported that regular exercise, particularly including the neuromuscular activation below the level of injury, can have a positive effect on the quality of life of an individual as well as on reducing the secondary consequences of cardio-metabolic diseases [52,53,54].

## 4. Conclusions

In this report, we demonstrate how the individual learned to manage his increased spasticity and restore partial control of movement under certain conditions. The increased spasticity was not downregulated through pharmaceutical methods, and conceivably played a role in the increased control of movement promoted by training-induced plasticity [45,55]. Regardless of the unchanged motor-complete ISNCSCI classification, this individual demonstrates motor control through a variety of movements when provided with an appropriate load and kinematic afferent feedback. The activity-based recovery training performed as part of his outpatient therapy promoted neuroplasticity below the level of injury and resulted in the unique and unlikely recovery demonstrated in this report. This report provides evidence of neuroplasticity occurring below the level of injury, regardless of the AIS classification and time since injury. The adoption of early, intense, task-specific and sensory-appropriate rehabilitation in the AIS-A population, followed by similar opportunities in the outpatient setting, could yield improved functional recovery for this group of individuals.

## Figures and Tables

**Table 1:** Exercises and body orientation.

Task	Orientation	Position	Variations
Push-ups	Prone	Bosu ball under hips; hands facing forward; shoulder width apart	Elbows out Elbows in
Shoulder flexion	Supine	Arms to side of trunk; legs extended	
Hip bridges	Supine	Arms to side of trunk; knees bent	On mat On Biodex
Trunk extension	Sitting	Trunk forward	Arms on lap Arms hanging
Hip/knee extension	Supine	Arms to side of trunk; hip and knee bent; pressure on plantar surface of foot	Single-leg (L and R) Single-leg with mini-shuttle
	Sitting	Sitting at edge of mat/chair	Single-leg with mini-shuttle Single-leg on Biodex

## Data Availability

The data presented in this study are available upon request from the corresponding authors. The data are not publicly available due to this being a single-case study.

## Supplementary Materials

The following supporting information can be downloaded at: https://www.mdpi.com/article/10.3390/jcm12216875/s1, Video S1: Voluntary movement and tasks performed by the individual with motor-complete SCI. Appendix S1. ISNCSCI evaluation scores were obtained at baseline during the screening process, and two additional ISNCSCI evaluation scores were obtained at prior timelines. In all of cases, the individual was classified as having a motor- and sensory-complete SPI (AIS-A).
