# Supplementary material for: Sensory Information Modulates Voluntary Movement in an Individual with a Clinically Motor- and Sensory-Complete Spinal Cord Injury: A Case Report
Source: J Clin Med. 2023 Oct 31;12(21):6875. doi: 10.3390/jcm12216875 (PMC10647542; doi:10.3390/jcm12216875)
Supplement: Supplementary file 1 [file jcm-12-06875-s001.zip › Apendix S1.pdf]

ISNCSCI Scores

Baseline

	RIGHT			LEFT		
	MOTOR	SENSORY		SENSORY		MOTOR
		LTR	PPR	LTL	PPL	
C2		2	2	2	2	
C3		2	2	2	2	
C4		1	0	1	1	
C5	0	0	0	0	0	0
C6	0	0	0	0	0	0
C7	0	0	0	0	0	0
C8	0	0	0	0	0	0
T1	0	0	0	0	0	0
T2		0	0	0	0	
T3		1	0	0	0	
T4		0	0	0	0	
T5		0	0	0	0	
T6		0	0	0	0	
T7		0	0	1	0	
T8		0	0	0	0	
T9		0	0	0	0	
T10		0	0	0	0	
T11		0	0	0	0	
T12		0	0	0	0	
L1		0	0	0	0	
L2	0	0	0	0	0	0
L3	0	0	0	0	0	0
L4	0	0	0	0	0	0
L5	0	0	0	0	0	0
S1	0	0	0	0	0	0
S2		0	0	0	0	
S3		0	0	0	0	
S4-5		0	0	0	0	
	0	6	4	8	5	0

Voluntary Anal Contraction: N

Deep Anal Pressure: N

7 months prior to Baseline

	RIGHT			LEFT		
	MOTOR	SENSORY		SENSORY		MOTOR
		LTR	PPR	LTL	PPL	
C2		2	2	2	2	
C3		2	2	2	2	
C4		2	2	2	2	
C5	0	1	0	0	0	0
C6	0	0	0	0	0	0
C7	0	0	0	0	0	3
C8	0	0	0	0	0	0
T1	0	0	0	0	0	0
T2		0	0	0	0	
T3		0	0	0	0	
T4		0	0	0	0	
T5		0	0	0	0	
T6		0	0	0	0	
T7		0	0	0	0	
T8		0	0	0	0	
T9		0	0	0	0	
T10		0	0	0	0	
T11		0	0	0	0	
T12		0	0	0	0	
L1		0	0	0	0	
L2	0	0	0	0	0	0
L3	0	0	0	0	0	2
L4	0	0	0	0	0	0
L5	0	0	0	0	0	0
S1	1	0	0	0	0	1
S2		0	0	0	0	
S3		0	0	0	0	
S4-5		0	0	0	0	
	1	8	6	6	6	6

Voluntary Anal Contraction: N

Deep Anal Pressure: N

3 months prior to baseline

	RIGHT			LEFT		
	MOTOR	SENSORY		SENSORY		MOTOR
		LTR	PPR	LTL	PPL	
C2		2	2	2	2	
C3		2	2	2	2	
C4		1	1	1	1	
C5	0	0	0	0	0	0
C6	0	0	0	0	0	0
C7	2	0	0	0	0	1
C8	0	0	0	0	0	0
T1	0	0	0	0	0	0
T2		0	0	0	0	
T3		0	0	0	0	
T4		0	0	0	0	
T5		0	0	0	0	
T6		0	0	0	0	
T7		0	0	0	0	
T8		0	0	0	0	
T9		0	0	0	0	
T10		0	0	0	0	
T11		0	0	0	0	
T12		0	0	0	0	
L1		0	0	0	0	
L2	0	0	0	0	0	0
L3	0	0	0	0	0	1
L4	0	0	0	0	0	0
L5	0	0	0	0	0	0
S1	1	0	0	0	0	1
S2		0	0	0	0	
S3		0	0	0	0	
S4-5		0	0	0	0	
	3	5	5	5	5	3

Voluntary Anal Contraction: N

Deep Anal Pressure: N
